# Trends in Early Growth Indices in the First 24 Months of Life in Uruguay over the Past Decade

**Published:** 2014-12

**Authors:** Isabel Bove, Cristina Campoy, Ricardo Uauy, Teresa Miranda, Florencia Cerruti

**Affiliations:** ^1^Uruguay Catholic University, Granada University, Spain; ^2^Department of Pediatrics, Granada University, Spain; ^3^Center of Excellence for Pediatric Research, EURISTIKOS; ^4^Institute of Nutrition and Food Technology, University of Chile, Chile; ^5^Department of Nutrition and Public Health Intervention Research, London School of Hygiene & Tropical Medicine, UK; ^6^Department of Biostatistics, Granada University, Spain; ^7^Uruguayan Network on Infant Feeding, Nutrition and Development (RUANDI)

**Keywords:** Breastfeeding, Low birthweight, Overweight, Rapid weight gain, Stunting, Uruguay

## Abstract

Early growth is an important indicator of health and wellbeing of children and a good predictor of adult health. The objective of this study was to examine trends and determinants of overweight and stunting among infants aged 0 to 23 month(s) over the past decade (1999-2011) in Uruguay. Data were used from four large representative samples of 11,056 infants aged 0-23 month(s), who attended public and private health services in 1999, 2003, 2007, and 2011, using a similar methodology. Linear regression analysis was used for assessing trends in early growth indices and binary logistic regression to estimate the probability of being stunted and overweight. Although prevalence of overweight fell from 12.5% (1999) to 9.5% (2011) and stunting from 13.6% to 10.9% respectively, both rates remained higher than expected. Low birthweight (LBW) was the main predictor of stunting [OR 6.5 (5.6-7.6)] and macrosomia of overweight [6.7 (5.3-8.3)]. We did not observe changes in LBW (7.8-8.8%) or macrosomia (5.9-6.7%) over the last decade. Boys showed increased chance of being overweight [OR 1.2 (1.04-1.3)]. Being stunted doubles the chances of being overweight [OR 2.5 (2.2-3.0)]. Overweight [OR 7.1 (6.1-8.3)], LBW [OR 13.2 (11.0-15.9)], and non-breastfed infants [OR 1.9 (1.7-2.1)] showed rapid weight gain. Uruguay has taken positive steps to decline the prevalence of stunting and overweight but both remain excessively high.

## INTRODUCTION

The last two decades experienced an accumulation of evidence, suggesting that the roots of the inequalities are embedded in early infancy. Conceptional period and the first years of life are determinants of health and wellbeing over the lifecycle. Infant's body-size and growth velocity during the early years of life are important indicators of wellbeing of children and are good predictors of health in adulthood (1). Optimal nutrition in infancy is essential for healthy growth and mental development. Evidence also highlights the role of foetal nutrition and early growth in the programming of disease management (2,3-8). Linear growth during infancy has been considered the best indicator of infants’ health (9). Stunted children have less learning capacity, less lean body mass, and they are at higher risk of being overweight (10-13). Low birthweight, followed by rapid weight gain during infancy, affects several components of the metabolic syndrome, increasing the risk of high blood pressure, altered glucose tolerance, overweight, central fat distribution as well as later obesity (14-17).

The objective of this study was to examine trends and determinants of overweight and stunting among infants aged 0 to 23 month(s) over the past decade (1999-2011) in Uruguay. During this period, the economy contracted and, after a few years, began to grow. As a consequence of the major financial crisis between 1999 and 2002, the country saw an increased population living under poverty conditions. While economic stability returned, renewed emphasis on social issues triggered a healthcare reform and the creation of the Ministry for Social Development. Efforts to promote breastfeeding and “good practices in nutrition” have been undertaken through the health services (18-20). Several years of strong economic growth reduced poverty from 39,6% (2004) to 13,7% (2011) and extreme poverty from 4.2% to 0.8%. The knowledge produced by this study can be useful to understand trends of overweight and stunting during different social and economic periods in Uruguay.

## MATERIALS AND METHODS

Data from four large representative samples of infants aged 0-23 month(s) in Uruguay were analyzed to address the objective of this study. The surveys were organized and ethically authorized by the Ministry of Public Health (MSP) and carried out by RUANDI with the support of UNICEF in 1999 (N=2,571), 2003 (N=2,783), 2007 (N=3,003), and 2011 (N=2,994). All surveys used a similar methodology. Only cross-sectional information was obtained. The survey results have been published by UNICEF (18-19).

Four probabilistic, multistage samples were selected, which were representative of the national and regional (capital and other provinces) levels. For each survey, a probabilistic sample was designed, which included the selection of provinces, health services, and children aged 0 to 23 month(s). The study was conducted in 58 health facilities located in the capital and 12 in 18 provinces of the country. Families were invited to participate in the study and asked to sign an informed consent prior to collection of data.

Sex, birthweight, and gestational age were taken from health records. Date of birth was also collected, and age was calculated. Information on infant-feeding practices was collected through a structured interview following recent WHO recommendations. Current anthropometric data were collected by appropriately-trained nutritionists. Children were weighed and measured without clothing or diapers, according to standardized techniques. Weight was measured by a scale with a precision of 0.1 kg. Horizontal length was measured with an infantometer with precision of 0.1 cm.

Using the WHO Child Growth Standards (2006), we estimated z-scores of current measures: length-for-age (LAZ), body mass index-for-age (BAZ), and weight-for-age (WAZ). Birthweight was also expressed as WAZ. WHO Anthro (version 3.2.2) was used in processing anthropometric data; 1.2% of measurements were excluded from the analyses, after being flagged as outliers according to the valid ranges accepted by WHO (WAZ <−6 or >5; LAZ <−6 or >6; BAZ <−5 or >5 z-scores) (WHO Anthro manual).

Infants with length-for-age <−2 SD (LAZ) below WHO reference population were classified as stunted and <−3 SD (LAZ) as severely stunted. Those with body mass index-for-age z-score (BAZ) >2 SD above WHO reference population were classified as overweight and >3 SD (BAZ) as obese.

Changes in SD scores between birth and current age were calculated for weight-for-age (WAZ at current age minus WAZ at birth). We considered indicating significant rapid weight gain when the difference from birth was greater than 0.67 z-scores (current WAZ minus WAZ at birth >0.67) (15,16).

### Statistical analysis

Descriptive statistics: mean, standard deviation (SD), median, minimum and maximum values were calculated. We used analysis of variance to test differences in z-scores over time and chi-square for categorical variables.

Trends in LAZ and BAZ over the years were assessed by linear regression analysis.

We included overweight and stunting as dependent variables. The year of the study was included as an independent variable. Binary logistic regression was carried out to estimate the probability of being stunted and overweight, controlling for birthweight, age, gender, breastfeeding duration, and socioeconomic level. The association of each explanatory variable was expressed as adjusted odds ratio (OR) with upper and lower bounds of 95% confidence interval.

Statistical significance level was defined by a p value (α) of <0.05. Management and analysis of data were performed using SPSS (version 15.0).

## RESULTS

We studied 5,710 (50.3%) boys and 5,641 (49.7%) girls aged less than 24 months (<6 months=2,898; 6-11 months=2,852; 12-17 months=2,790, and 18-23 months=2,811). We observed a high prevalence of overweight [11.3% (10.7-11.9%)] and stunting [13.3% (12.7-13.9%)] over the last decade in Uruguay (Table 1).

**Figure 1.**
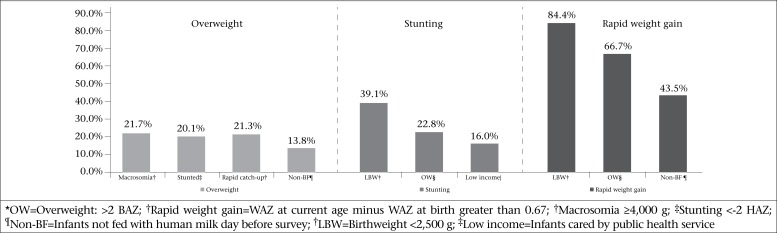
Higher rates of overweight^*^, stunting^‡^ and rapid weight-gain^†^ in infants aged less than 24 months

Low birthweight (LBW) was the main predictor of being stunted [OR 6.5 (5.6-7.6)] and macrosomia of being overweight [OR 6.7 (5.3-8.3)] (Table 2 and Figure 1). During the study period, we did not observe changes in low birthweight (7.8-8.8%) or in macrosomia (5.9-6.7%) (Table 1).

**Table 1.** Prevalence of low birthweight (LBW)†, macrosomia†, overweight (OW)§, and stunting‡ in boys and girls aged 0 to 23 month(s) in Uruguay in 1999, 2003, 2007, and 2011

LBW†	LBW n (%)*	95% CI*	Boys n (%)	Girls n (%)	p value	
1999	199 (7.7)	6.7-8.7	104 (7.9)	95 (7.6)	0.41	
2003	223 (8.1)	7.3-9.3	224 (8.3)	112 (8.6)	0.33	
2007	248 (8.3)	7.3-9.3	111 (7.2)	138 (9.5)	0.01	
2011	260 (8.7)	7.3-9.7	126 (8.3)	134 (9.1)	0.23	
Total	930 (8.3)	7.8-8.8	452 (7.8)	478 (8.7)	0.04	
Macrosomia†	n (%)*	95% CI*	Boys n (%)	Girls n (%)	p value	
1999	169 (6.6)	5.6-7.6	114 (8.7)	155 (4.4)	<0.001	
2003	176 (6.5)	5.6-7.4	131 (9.4)	45 (3.4)	<0.001	
2007	181 (6.0)	5.2-7.0	120 (7.8)	61 (4.2)	<0.001	
2011	181 (6.1)	5.2-7.0	99 (6.5)	82 (5.6)	0.16	
Total	708 (6.3)	5.9-6.7	464 (8.0)	244 (4.5)	<0.001	
OW§	n (%)*	95% CI*	Boys n (%)	Girls n (%)	p value	Obesity§ n (%)
1999	318 (12.5)	11.2-13.8	183 (14.1)	135 (10.9)	0.01	98 (3.9)
2003	382 (14.1)	12.8-15.4	219 (15.8)	163 (12.4)	0.01	96 (3.6)
2007	279 (9.3)	8.3-10.3	161 (10.4)	118 (8.2)	0.02	53 (1.8)
2011	279 (9.5)	8.4-10.6	145 (9.7)	134 (9.2)	0.33	45 (1.5)
Total	1,258 (11.3)	10.7-11.9	708 (12.4)	550 (10.1)	<0.001	292 (2.6)
Stunting‡	n (%)*	95% CI*	Boys n (%)	Girls n (%)	p value	Severe stunting‡ n (%)
1999	368 (13.6)	12.3-14.9	236 (18.5)	132 (10.6)	<0.001	130 (5.2)
2003	459 (16.4)	15.0-17.8	279 (20.0)	180 (13.8)	<0.001	154 (5.7)
2007	338 (11.3)	10.2-12.4	201 (13.0)	137 (9.5)	<0.001	97 (3.2)
2011	320 (10.9)	9.8-12.0	177 (11.9)	142 (9.8)	0.04	78 (2.7)
Total	1,485 (13.3)	12.7-13.9	893 (15.7)	591 (10.9)	<0.001	459 (4.1)

*Number of cases observed; percent valid cases; 95% confidence interval; p=Level of significance according to χ^2^;

†LBW=Birthweight <2,500 g;

†Macrosomia birthweight ≥4,000 g;

§OW=Overweight >2 BAZ; Obesity >3 BAZ;

‡Stunting <−2 HAZ; Severe stunting <−3 HAZ

Both overweight and stunting were significantly higher in boys than girls; boys showed an increased chance of being overweight [OR 1.2 (1.04-1.3)] as well as being stunted [OR 1.6 (1.4-1.8)] (Table 1 and 2).

**Figure 2.**
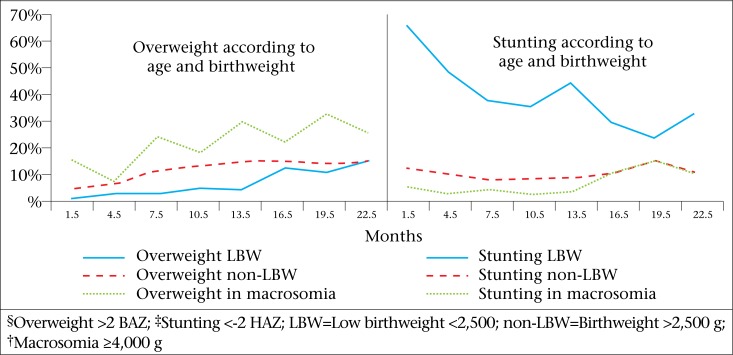
Overweight^§^ and stunting^‡^ in low-birthweight (LBW) infants non-low-birthweight (non-LBW) infants and macrosomia

Infants aged <6 months were three times more likely to be stunted than infants aged 6 to 23 months. On the other hand, the risk of being overweight increased with age, doubling when children were aged >6 months [OR 2.4 (1.9-3.0)] (Table 2 and Figure 2).

When the infants showed rapid weight gain (current WAZ minus WAZ at birth >0.67), they increased probability of being overweight [OR 7.1 (6.1-8.3)]. We observed the highest risk of rapid weight gain among LBW [OR 13.2 (11.0-15.9)] and non-breastfed [OR 1.9 (1.7-2.1)] infants. LBW infants showed so rapid postnatal weight gain that they reached the overweight prevalence of non-LBW after 20 months of age as can be seen in Figure 2.

Stunting and overweight were closely associated. Being stunted doubled the chance of being overweight [OR 2.5 (2.2-3.0)] (Table 2 and Figure 1).

Table 3 shows the trends in body mass index-for-age (BAZ) and length-for-age (LAZ) over the last decade. Mean LAZ increased by +0.016 z-score, and mean BAZ decreased by −0.018 per year studied.

**Table 2.** Relative predictors of overweight§, stunting‡, and rapid weight-gain†† in 1999, 2003, 2007, and 2011 in Uruguay

Overweight§ 1,258 (11.3%)	n (%)*	p value	OR (95% CI)
Year 2003	385 (14.3)	<0.001	1.4 (1.2-1.7)
Year 1999	327 (12.6)	0.020	1.2 (1.04-1.5)
Rapid weight gain††	799 (21.3)	<0.001	7.1 (6.1-8.3)
Macrosomia†	151 (21.7)	<0.001	6.7 (5.3-8.3)
Stunting‡	270 (20.1)	<0.001	3.1 (2.6-3.6)
6 to 23 months	686 (14.5)	<0.001	2.4 (1.9-3.0)
Non-BF¶	644 (13.8)	0.024	1.2 (1.02-1.3)
Boys	684 (12.6)	0.010	1.2 (1.04-1.3)
Stunting‡ 1,485 (13.3%)	n (%)*	p value	OR (95% CI)
Year 2003	461 (17.1)	<0.001	1.5 (1.2-1.7)
Year 1999	370 (14.7)	0.006	1.3 (1.1-1.5)
LBW†	337 (39.1)	<0.001	6.5 (5.6-7.6)
OW§	270 (22.8)	<0.001	2.5 (2.2-3.0)
Low income‡	990 (16.2)	<0.001	2.1 (1.8-2.4)
Boys	834 (15.4)	<0.001	1.6 (1.4-1.8)
<6 months	396 (14.7)	<0.001	1.3 (1.1-1.6)
Rapid weight gain†† 3,764 (34,3%)	n (%)*	p value	OR (95% CI)
LBW†	726 (84.4)	<0.001	13.2 (11.0-15.9)
OW§	799 (66.7)	<0.001	5.5 (4.8-6.3)
Non-BF¶	2,072 (43.5)	<0.001	1.9 (1.7-2.1)

*Number of cases observed; percent valid cases;

**p=Level of significance; OR=Odds ratios obtained from logistic regression, adjusted models=EXP (β). 95% confidence interval for EXP (β);

§OW=Overweight >2 BAZ;

†Macrosomia ≥4,000 g;

‡Stunting <−2 HAZ;

††Rapid weight gain WAZ at current age minus WAZ at birth greater than 0.67 z-scores;

¶Non-BF=Infants not fed with human milk day before survey;

†LBW=Birthweight <2,500 g;

‡Low income=Infants cared by public health service

Prevalence of overweight fell from 12.5% in 1999 to 9.5% in 2011 and stunting from 13.6% to 10.9%. Particularly noticeable was the change in obesity (3.9% to 1.5%) and severe stunting (5.2% to 2.7%). In 2003, we observed a rise in the prevalence of stunting from 13.6% to 16.4% and overweight from 12.5% to 14.1% compared to 1999 (Table 1).

## DISCUSSION

In spite of having a strong commitment to democracy, with the most equitable distribution of income in Latin America, the results of this study suggest that Uruguay needs to rethink new strategies to improving physical growth in early infancy for optimal health and wellbeing through the course of life.

**Table 3.** Body mass index-for-age (BAZ) and length-for-age (LAZ) trends over years among infants aged 0 to 23 month(s) in Uruguay, 1999, 2003, 2007, and 2011

Parameter	Mean±SD	Regression coefficient*	95% CI	p value
BAZ	0.56±1.22	-0.018	-0.023 to −0.013	<0.001
HAZ	-0.59±1.36	0.016	0.01 to 0.022	<0.001

*Regression coefficients represent the amount of dependent variable and changes when the corresponding independent variable changes by 1 unit

We confirm that underweight was not a problem but stunting and overweight rates remained higher than expected in the context of Uruguay. Despite robust income growths and important decreases in poverty, low birthweight (LBW) rate did not reduce, and prevalence of stunting fell only 2.7% over the last decade. LBW rate and prevalence of stunting in 2011 were higher than in Costa Rica, Cuba, and Chile (21-23). An increase in the prevalence of stunting in 2003 was consistent with the worst socioeconomic and financial crisis in Uruguay in the past century.

As has been reported by other authors, our results showed that boys were more vulnerable to poor growth compared to girls (21,24-25). Male students in public school system in Uruguay are those with the highest prevalence of stunting (20) and the poorest educational performance (39).

In the present study, the probability of being overweight among stunted infants doubled, indicating the recognized association between poverty and obesity as well as the double burden in the social, economic, and healthcare systems (12,13,27-28)

Prevalence of overweight, despite showing positive steps towards decline, is still too high in Uruguay (2011: 8.4-10.6%). Special attention should be given to avoid fast catch-up weight gain in low-birthweight infants (5,6,8,10,17,37,38). In the present study, non-breastfed infants showed an increased risk of being overweight and especially increased chance of fast catch-up weight gain (33,34).

To improve linear growth and, at the same time, to address measures to avoid excessive weight gain, Uruguay should continue encouraging exclusive breastfeeding, particularly to LBW infants as well as improving complementary food practices that provide an adequate micronutrient supply after 6 months of age (21,25,30,32-37).

### Strengths and limitations

The main limitation of this study is the fact that we analyzed cross-sectional data with only one measure per child; so, we only could describe association and could not establish causal relationship. The strength of this study is the robust sample-sizes analyzed as well as that children included in the analysis were from different socioeconomic families. On the other hand, there was little information about Uruguayan early growth indices before this study.

### Conclusions

Uruguay has taken positive steps to decline the prevalence of stunting and overweight. Nevertheless, both remain excessively high and, according to our results, new strategies need to be thought of.

## ACKNOWLEDGEMENTS

The four surveys were financed by UNICEF of Uruguay. This analysis is part of the first author´s thesis to fulfill the requirements for a doctorate, and it was supported by Monesia Group, Granada University.

We would like to acknowledge Alvaro Arroyo for his constant help; Cristina Lustemberg, Ximena Moratorio, Mara Castro, Jorge Quian, Cecilia Muxi for trust, diffusion, and providing logistics to carry out these surveys; Valentina Muxi for excellent field work supervision; Walter Álvarez for sample selection; Graciela Romano, Monica Márquez and Jacqueline Lucas for their assistance in interviewers’ training. We thank UNICEF and the participating families for making these studies possible.

**Competing interests:** The authors declare no conflict of interest.
